# 708 Chemokine Signature Pattern in Toxic Epidermal Necrolysis: Diagnostic and Therapeutic Relevance

**DOI:** 10.1093/jbcr/irae036.253

**Published:** 2024-04-17

**Authors:** Andrew Anklowitz, Michael Quinn, Justin Hale, Dana Poloni, Sanjiv Kumar, Marcus Aranda, Balakrishna Prasad

**Affiliations:** Joseph M. Still Research Foundation, Inc., Augusta, GA; Eisenhower Army Medical Center, Ft. Gordon, GA; Joseph M. Still Research Foundation, Inc., Augusta, GA; Eisenhower Army Medical Center, Ft. Gordon, GA; Joseph M. Still Research Foundation, Inc., Augusta, GA; Eisenhower Army Medical Center, Ft. Gordon, GA; Joseph M. Still Research Foundation, Inc., Augusta, GA; Eisenhower Army Medical Center, Ft. Gordon, GA; Joseph M. Still Research Foundation, Inc., Augusta, GA; Eisenhower Army Medical Center, Ft. Gordon, GA; Joseph M. Still Research Foundation, Inc., Augusta, GA; Eisenhower Army Medical Center, Ft. Gordon, GA; Joseph M. Still Research Foundation, Inc., Augusta, GA; Eisenhower Army Medical Center, Ft. Gordon, GA

## Abstract

**Introduction:**

Toxic Epidermal Necrolysis (TEN) is a delayed hypersensitivity reaction to some pharmacological agents. Common drugs involved include antibiotics, anticonvulsants, and allopurinol. The pathophysiology of TEN is not well understood. The mortality of TEN is upwards of 50%, and more research is needed to help understand this disease to develop diagnostic assays and potential targeted treatments. This study aimed to identify the signature pattern of cytokine/chemokine response to TEN.

**Methods:**

To distinguish the immune response to skin injury from pathogenic immune activation leading to TEN, we studied the cytokine profile in burn and TENS patients during the first week of hospitalization. We analyzed daily plasma samples from 25 burn and 9 TEN patients using the Luminex Multiplex assay platform.

**Results:**

Several pro-inflammatory cytokines were increased in both burns and TENS patients. However, three chemokines were significantly elevated in almost all TENS patients analyzed compared to the burn cohort: CXCL9, CXCL10, and CXCL11. This response was sustained for the one week of hospital stay.

**Conclusions:**

The chemokine/cytokine signature response appears to be involved in the pathophysiology of TEN. This signature response could be used to develop in-vitro diagnostic assays to identify causative agents and to develop therapeutic drugs targeting the pathologic response in TENS.

**Applicability of Research to Practice:**

Identifying and determining the significance of specific chemokines that remain significantly elevated helps serve as preliminary data to justify a larger, clinical study that may help in understanding this disease process better, to help develop diagnostic assays and to help develop potential targeted treatments.

